# Relationship between Thyroid CT Density, Volume, and Future TSH Elevation: A 5-Year Follow-Up Study

**DOI:** 10.3390/life13122303

**Published:** 2023-12-06

**Authors:** Tomohiro Kikuchi, Shouhei Hanaoka, Takahiro Nakao, Yukihiro Nomura, Takeharu Yoshikawa, Md Ashraful Alam, Harushi Mori, Naoto Hayashi

**Affiliations:** 1 Department of Computational Diagnostic Radiology and Preventive Medicine, The University of Tokyo Hospital, Tokyo 113-8655, Japanaalam@g.ecc.u-tokyo.ac.jp (M.A.A.);; 2 Department of Radiology, School of Medicine, Jichi Medical University, Tochigi 329-0498, Japan; 3 Department of Radiology, The University of Tokyo Hospital, Tokyo 113-8655, Japan; 4 Center for Frontier Medical Engineering, Chiba University, Chiba 263-8522, Japan

**Keywords:** hypothyroidism, segmentation, thyroid density, thyroid-stimulating hormone, thyroid volume

## Abstract

This study aimed to explore the relationship between thyroid-stimulating hormone (TSH) elevation and the baseline computed tomography (CT) density and volume of the thyroid. We examined 86 cases with new-onset hypothyroidism (TSH > 4.5 IU/mL) and 1071 controls from a medical check-up database over 5 years. A deep learning-based thyroid segmentation method was used to assess CT density and volume. Statistical tests and logistic regression were employed to determine differences and odds ratios. Initially, the case group showed a higher CT density (89.8 vs. 81.7 Hounsfield units (HUs)) and smaller volume (13.0 vs. 15.3 mL) than those in the control group. For every +10 HU in CT density and −3 mL in volume, the odds of developing hypothyroidism increased by 1.40 and 1.35, respectively. Over the course of the study, the case group showed a notable CT density reduction (median: −8.9 HU), whereas the control group had a minor decrease (−2.9 HU). Thyroid volume remained relatively stable for both groups. Higher CT density and smaller thyroid volume at baseline are correlated with future TSH elevation. Over time, there was a substantial and minor decrease in CT density in the case and control groups, respectively. Thyroid volumes remained consistent in both cohorts.

## 1. Introduction

Primary hypothyroidism, which is a common hormonal imbalance marked by high thyroid-stimulating hormone (TSH) levels, has two main types: subclinical, with normal free thyroxine (T4) levels, and overt, with below-normal T4 levels [1]. The prevalence of hypothyroidism ranges from 4% to 15%, is higher among women, and increases with age [2,3,4]. It has diverse symptoms, ranging from life-threatening to asymptomatic, and these include fatigue, cold sensitivity, weight gain, constipation, voice changes, and dry skin. Additionally, hypothyroidism can affect the cardiovascular system and bones [1,5]. Its diagnosis is impeded by its diverse and often non-specific symptoms, which increases the risk of undetected cases. Accordingly, it is important to increase knowledge for the identification of high-risk groups and early diagnosis of hypothyroidism.

Ultrasound is the most common imaging modality for thyroid assessment; however, computed tomography (CT) imaging allows unique assessments since it can reflect the iodine content of the thyroid gland [6]. Additionally, the thyroid is often included in the imaging range of routine chest CTs; therefore, its size and CT density are commonly assessed during standard radiological interpretations. Cross-sectional studies have shown that thyroid CT densities in conditions such as chronic thyroiditis and hypothyroidism typically range from 36 to 81 Hounsfield units (HUs), which is are significantly lower than the normal range of 80–120 HU [7,8,9,10]. This decreased CT density can be attributed to the replacement of thyroid follicular cells, infiltration of inflammatory cells, and subsequent fibrosis [7,8,11,12,13].

However, there remain no large-scale longitudinal studies on thyroid CT densities; accordingly, the baseline characteristics and changes associated with the onset of hypothyroidism remain unclear. Furthermore, in cases of excessive iodine intake, the thyroid gland increases iodine uptake, which increases the risk of the future onset of chronic thyroiditis and thyroid dysfunction [14,15]. Accordingly, iodine-rich intake may result in increased thyroid CT densities prior to the onset of the aforementioned conditions. Although comprehensive studies on the relationship between dietary iodine exposure and thyroid CT densities are lacking, it is known that excessive iodine exposure through medications like amiodarone can lead to an increase in thyroid densities [16,17]. Additionally, there are scarce longitudinal data regarding thyroid volume.

For improved thyroid assessment via CT, which may facilitate the risk evaluation and early diagnosis of hypothyroidism, a comprehensive longitudinal analysis of CT densities and volumes is essential. Our institution has a large database of whole-body CT scans and time series data from blood tests performed as part of the annual medical checkup program. Although the manual measurement of CT density and volumes in numerous images was previously labor-intensive, advances in deep learning-based segmentation techniques have now provided an efficient solution [18,19]. These techniques enable precise pixel-level extraction in CT images, which simplifies the acquisition of quantitative data. By leveraging this database with deep learning-driven segmentation techniques, this study aimed to examine the correlations between initial CT characteristics and the subsequent emergence of hypothyroidism. Further, we aimed to examine alterations in imaging indicators from baseline values among individuals who subsequently developed hypothyroidism.

## 2. Materials and Methods

### 2.1. Dataset and Study Design

This retrospective study was approved by the ethics review board of our institution. Our comprehensive database consolidates data, including sex, age, and blood test results such as TSH and T4 levels, as well as whole-body CT images of healthy individuals who participated in our medical cancer screening program. In this database, we tracked information regarding the above items for individuals who first visited the program between November 2006 and October 2011 for up to 6 years. Written informed consent was obtained from all participants for the use of their screening data for research purposes.

In our study, cases without a record of a visit from 5 to 6 years after the initial consultation were defined as censored at the time of their last visit. After excluding individuals with missing information, without follow-up visits, with previous thyroid surgery, and with elevated TSH levels at the initial visit, the remaining individuals were defined as the source cohort. Subsequently, we performed a nested case–control exploration, with the focal outcome being the onset of elevated TSH levels. In this cohort, participants who exceeded the upper limit of the TSH level during the course of the study were defined as the case group. Among individuals without elevated TSH levels, those with available follow-up data for >5 years were included in the control group. We examined differences in the baseline characteristics between the case and control groups. Additionally, we checked for significant changes in CT density and volume between the first visit and the end of follow-up (i.e., when the TSH level exceeded the upper limit for the case group and at the last visit for the control group).

### 2.2. Image Acquisition and Measurements

Whole-body CT scans in our database were performed using single-type scanners (Discovery ST Elite, GE Healthcare, Waukesha, WI, USA) without a contrast medium. The CT images were acquired with the participants’ arms down using the following parameters: tube voltage, 120 kV; field of view, 500 mm; matrix size, 512 × 512; and voxel size, 0.98 × 0.98 × 1.25 mm. Using CT images, the CT densities and volumes of the thyroid gland were calculated using the deep learning method described below.

**Figure 1:**
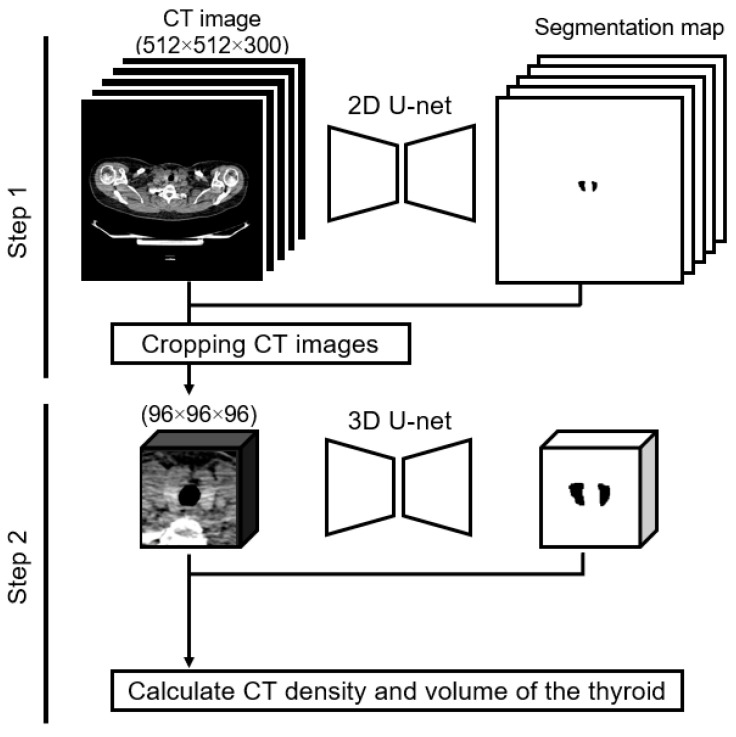
Two-step method of calculating thyroid CT density and thyroid volume. Step 1: the input is a two-dimensional whole-body CT image, and the output is a label image. We created a cropped CT image of 96 × 96 × 96 pixels based on the label image. Step 2: the input is the cropped three-dimensional CT image, and the output is the label image. We calculated the thyroid CT density and volume using the input and output in Step 2.

Dual U-net architectures were used to attain a precise segmentation map of the thyroid gland. The U-net design, which is renowned for its symmetric U-shaped structure fortified with lateral skip connections, has gained significant traction in the realm of medical image semantic segmentation [19,20]. Our approach entailed a two-tiered process: we initiated coarse thyroid location prediction via a two-dimensional U-net, followed by more refined segmentation facilitated by a cropped three-dimensional U-net (Figure 1). The implementation was conducted using Python 3.8.13 (accessed on 3 December 2023, https://docs.python.org/3.8/) and PyTorch 1.11.0 (accessed on 3 December 2023, https://pytorch.org/docs/1.11/). The subsequently derived segmentation maps were instrumental in computing the thyroid CT density and volume for each participant. The computed CT density represented the average CT density across the entirety of the segmented thyroid, whereas the volume was ascertained by multiplying the voxel count within the segmented map by the dimensions of an individual voxel.

As part of our preliminary assessments, the efficacy of our methodological implementation was gauged. When trained on a dataset comprising 107 cases (training cohort, 85; validation cohort, 22)—these scans were captured using a consistent CT scanner, though they were not incorporated into our primary analysis dataset—the model exhibited a commendable Dice similarity coefficient [21] of 0.93. This demonstrated that our approach could achieve high-quality semantic segmentation.

### 2.3. Statistical Analysis

Differences in baseline distributions between the case and control groups were examined using the chi-square test for sex and the Mann–Whitney U test for age, TSH level, T4 level, CT density, and volume. Scatter plots of CT density at baseline and volume were created for visualization. Logistic regression analysis was performed to examine the association between CT imaging features (CT density and volume) and new abnormalities. Sex, age, TSH level, and T4 level at baseline were used as covariates. The Wilcoxon signed-rank test was used to assess whether or not the CT density and volume changed from baseline to follow-up end within the groups. Between-group comparisons of these changes were not performed since there was no appropriate compensation for the different follow-up periods in the two groups. Scatter plots were generated for changes in CT densities and TSH levels. Subsequently, ordinary least squares (OLS) regression analysis was employed to draw regression lines on these plots, quantifying the relationship between changes in CT densities and TSH levels. All statistical analyses were performed using the JMP Pro 17.0.0 software (JMP Statistical Discovery LLC, Cary, NC, USA). Statistical significance was defined as *p* < 0.05.

## 3. Results

**Figure 2:**
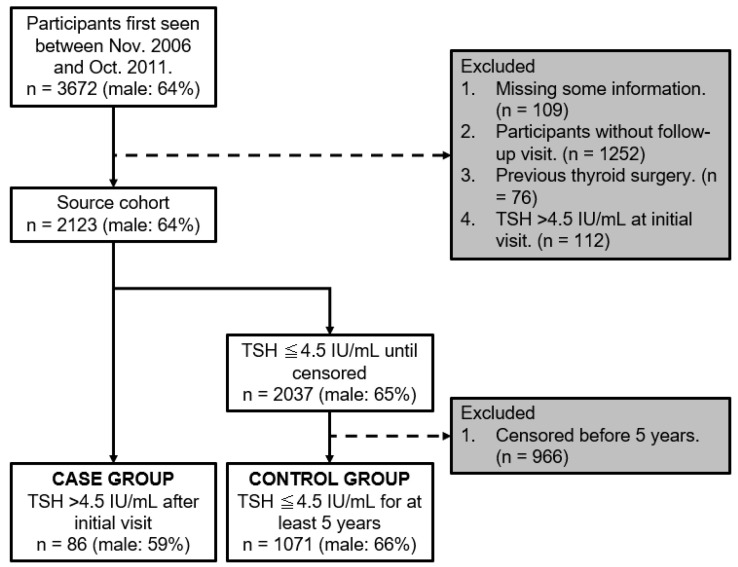
Flowchart of participants.

Figure 2 shows a flowchart of the participants. Between November 2006 and October 2011, 3672 participants were initially included. Among them, participants were excluded in accordance with the exclusion criteria; 109 were missing crucial information, 1252 did not attend follow-up visits, 76 had a history of thyroid surgery, and 112 presented with a baseline TSH level > 4.5 IU/mL. Subsequently, the source cohort stood at 2123 participants. Among them, 2037 participants had a TSH level ≤ 4.5 IU/mL until censored, with 966 being excluded for being censored before a 5-year span. Subsequently, participants were segmented into two main groups. The case group comprised 86 participants with a TSH level > 4.5 IU/mL after their initial visit, while the control group comprised 1071 participants who maintained a TSH level of ≤4.5 IU/mL for ≥5 years.

Table 1 summarizes the distribution of each variable per group at baseline. The participants in the case group tended to be older (median: 61 vs. 56 years; *p* < 0.01), and had higher TSH levels (median: 2.80 vs. 1.30 IU/mL; *p* < 0.01), lower T4 levels (median: 1.08 vs. 1.12 ng/dL; *p* < 0.01), higher CT densities (median: 89.8 vs. 77.5 HU; *p* < 0.01), and lower volumes (median: 13.0 vs. 15.1 mL; *p* < 0.01) than those in the control group at baseline. The proportion of male participants was non-significantly lower in the case group than in the control group (male%: 59% vs. 66%; *p* = 0.22).

**Figure 3:**
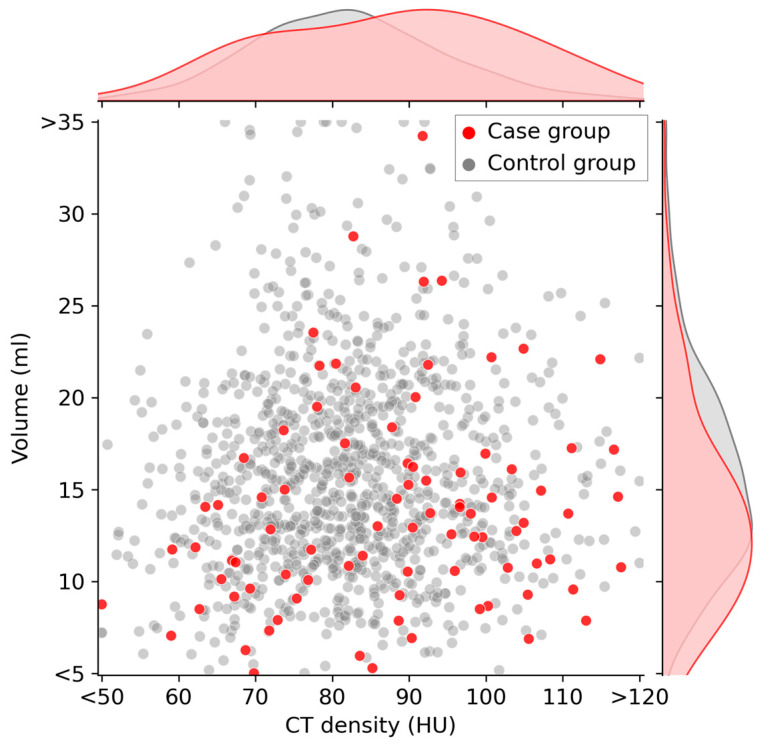
Scatter plot of thyroid CT density and thyroid volume at baseline. Participants in the case group are indicated by red dots on the graph. These red dots tend to be located on the lower-right quadrant of the graph. The curves along the top and right side of the plot area represent the kernel density estimate curves for each variable.

Figure 3 illustrates a scatter plot highlighting the relationship between CT density (HU) and volume (ml) at baseline. The case and control groups are distinctly represented by red and gray dots, respectively. Upon observation, it becomes apparent that the red data points of the case group tend to shift slightly toward the lower-right quadrant, indicating a lower volume and higher CT density compared with those indicated by the gray data points of the control group.

For every 10 HU increase in CT density, the non-adjusted odds ratio for new-onset TSH elevation was 1.40, with a 95% confidence interval (CI) of 1.16–1.67 (*p* < 0.01). Similarly, for a 3 mL reduction in volume, the odds ratio was 1.26 (95% CI: 1.11–1.59; *p* < 0.01). After adjusting for baseline factors such as sex, age, TSH level, and T4 level, the odds ratios for a 10 HU increase in CT density and 3 mL decrease in volume were 1.44 (95% CI: 1.17–1.77; *p* < 0.01) and 1.33 (95% CI: 1.11–1.59; *p* < 0.01), respectively. Despite these adjustments, the significance remained unchanged. These findings are detailed in Table 2.

In the case group, during a median observation span of 2.1 years (interquartile range: 1.9–3.4 years), there was a notable reduction in CT density. The average decrease in CT density was −8.4 HUs, with a range from −17.1 to −1.1 HUs; further, this change was statistically significant (*p* < 0.01), as indicated in Table 3. Contrastingly, the shift in volume was minimal and not statistically significant, with an average change of −0.1 mL and a range from −1.9 to 1.2 mL (*p* = 0.23). Similarly, among 870 participants in the control group without any TSH elevations over a 5-year period, there was a statistically significant decrease in CT density, with an average of −2.9 HUs and ranging from −8.5 to 1.8 HUs (*p* < 0.01). However, similar to the case group, the volume change was negligible and not statistically significant, with an average alteration of −0.1 mL, within a range of −1.4 to 1.2 mL (*p* = 0.29).

**Figure 4:**
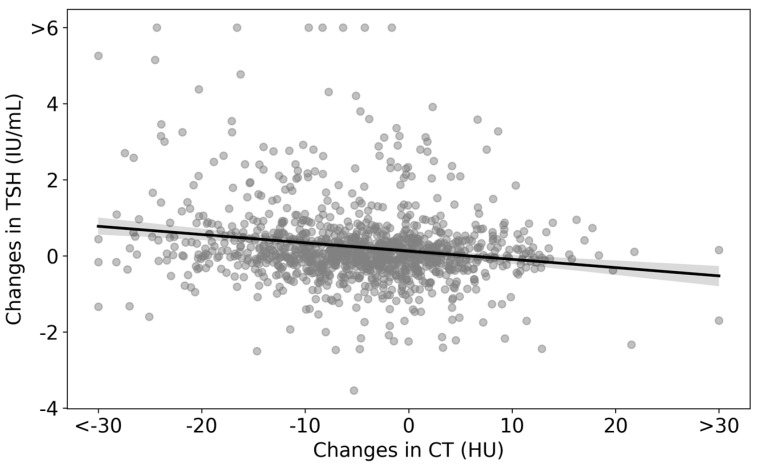
Scatter plot of changes in CT densities and TSH levels in the analyzed cohort. The scatter plot represents the differences between the values at the end of the follow-up and the initial consultation for both CT densities and TSH. The regression line is given by ΔTSH = −0.022 × (ΔCT density) + 0.1240.

Changes in CT densities and TSH levels are visualized as a scatter plot in Figure 4. The regression line is represented by the equation ΔTSH = −0.022 × (ΔCT density) + 0.1240, which indicates an inverse relationship between changes in CT densities and TSH. The statistical analysis yielded a *p*-value of less than 0.01 for the ΔCT density coefficient (Table 4).

## 4. Discussion

Our findings demonstrated that higher CT densities and smaller thyroid volumes at baseline are associated with subsequent elevations in TSH levels. The case group experienced a significant decrease in CT density, whereas the control group experienced only a minor decrease, with both groups maintaining stable volumes over time. To our knowledge, this is the first study to perform a comprehensive longitudinal analysis regarding the relationship between TSH levels and CT imaging characteristics of the thyroid gland.

Recent advancements in deep learning technology have significantly impacted the field of medical image analysis [19,22]. The segmentation technique employed in this study, which uses deep learning, involves labeling each pixel in an image to represent the characteristics of the corresponding region [20,23]. By utilizing tens to hundreds of annotated images for training neural networks, we can automate the extraction of regions of interest. This automation has drastically increased the efficiency of tasks that traditionally required considerable effort for each case at imaging workstations. Furthermore, apart from identifying regions, these networks can provide various quantitative data depending on the applied imaging modality. These quantitative data include volume measurements, CT density, signal intensity for magnetic resonance imaging (MRI), or a standardized uptake value (SUV) for positron emission tomography (PET) [24,25,26]. Therefore, deep learning-enhanced image segmentation is a crucial and valuable fundamental technique for large-scale image analysis. This segmentation technique and the large database facilitated our large-scale analysis and provided new insights into this field. Although several studies have employed deep learning for thyroid analysis, the current primary focus appears to be on using ultrasound to detect thyroid nodules or differentiate between benign and malignant nodules [27,28,29], with CT studies being relatively limited. However, the thyroid is often included in the upper bound of standard chest CT scans, suggesting a potentially large dataset for analysis. Accordingly, this approach is expected to receive increasing attention, thus leading to more detailed research, including longitudinal studies. Moreover, using the quantitative values of regions of interest, this approach can provide explanatory or dependent variables for epidemiological studies, similar to other clinical information, which may further invigorate epidemiological research, including image feature analysis.

In our observations, we observed notable differences between the case and control groups. Compared with the control group, the case group tended to have higher thyroid CT density (median: 89.8 HU for the case group; 77.5 HU for the control group; *p* < 0.01) and smaller thyroid volumes (median: 13.0 mL for the case group; 15.1 mL for the control group; *p* < 0.01) at baseline. Moreover, when the TSH level exceeded the upper limit, there was a large decrease in thyroid CT densities (median: −8.4 HU) but almost no change in thyroid volume (median: −0.1 mL) in the case group; similarly, in the control group, there was a slight but significant decrease in CT densities (median: −2.9 HU), but almost no change in volume (median: −0.1 mL). The observed marginally lower proportion of male participants in the case group, despite not reaching statistical significance, is consistent with previous findings. Numerous studies have demonstrated that the incidence of hypothyroidism tends to be higher among women and escalates with advancing age [2,4]. Moreover, the higher baseline TSH level in the case group is reasonable since it comprised participants whose TSH levels were in the process of rising at baseline. A high CT density and low volume of the thyroid was associated with new-onset TSH level elevation even after adjusting for other baseline information (i.e., sex, age, TSH levels, and T4 levels) in logistic regression (Table 2). The higher CT densities in the case group than those in the control group could be attributed to iodine intake. Previous studies have suggested that high iodine intake leads to increased TSH levels and thyroid iodine uptake, which may be reflected as increased CT densities [6]. Furthermore, excessive iodine intake can trigger chronic thyroiditis and hypothyroidism [14,15]. Therefore, high thyroid CT densities could be indicative of excessive iodine intake, with these cases potentially developing chronic thyroiditis or hypothyroidism. The significant decrease in CT densities in the case group could be attributed to the onset of chronic thyroiditis. Unfortunately, our study could not verify this hypothesis given the lack of dietary and thyroid peroxidase (TPO) antibody data. Nonetheless, our findings provide a basis for more detailed future research on iodine exposure, CT findings, and thyroid function. Regarding thyroid volume, a previous cohort study using ultrasonography indicated that smaller thyroid sizes are associated with the development of hypothyroidism [30], which is consistent with our findings. However, the underlying mechanism of this relationship remains unclear, and further research is warranted. Another important point to note is that when examining the endpoint of the follow-up, only a slight between-group difference was observed in the CT densities. This suggests that, in the context of predicting elevated TSH levels, relying solely on assessing thyroid CT density at a single time point may not be sufficient. Therefore, it is crucial to monitor changes in thyroid CT density over time. If a notably low thyroid CT density is incidentally observed, the measurement of TSH should be considered.

In the course of this study, we observed nuanced shifts in CT density (−2.9 [−8.5–1.8] HU over a span of 5.5 [5.4–5.7] years) and volume (−0.1 [−1.4–1.2] mL across the same time frame) within the control groups. Our interpretation of these results leads us to postulate that such changes may indeed signify “the typical 5-year aging transformations of the thyroid”, as captured via CT imaging. Moreover, we observed an inverse relationship between changes in CT densities and changes in TSH levels (Figure 4 and Table 3). These findings could inform future longitudinal imaging studies and routine CT interpretations related to thyroid health. For example, these results can serve as a foundational reference for interpreting reduced CT densities and potential enlargement in conditions such as chronic thyroiditis or identifying thyroid atrophy stemming from side effects of oncological treatments [30,31,32]. Taken together, we believe that our study provides fundamental information for correctly interpreting changes in thyroid imaging findings.

Building on our findings, we envisage potential developments in the following research topics. First, we consider whether or not it is feasible to predict elevations in TSH levels by observing changes in thyroid CT density. We observed a pronounced decline in CT densities in the case group; however, it remains unclear whether this decrease is a precursor to or a consequence of TSH elevation. By examining whether or not alterations in CT densities are harbingers of functional changes, we might be able to establish a system capable of preemptively flagging TSH increases during CT scans not originally intended for thyroid evaluation. Secondly, a detailed examination is necessary regarding the correlation between iodine intake, including from diet and medications, and histopathological changes, as well as their correspondence with imaging. The integration of such multidisciplinary information will contribute to a further elucidation of the mechanisms from normal to pathological states. Additionally, a holistic assessment incorporating clinical symptoms is warranted. Hypothyroidism, while acknowledged as a significant clinical state, is also the subject of studies suggesting that aging-related TSH elevations may not hold clinical significance [33,34]. Differentiating clinically meaningful TSH elevations from those attributable to aging presents a challenge. Nevertheless, we posit that an integrated analysis, encompassing imaging characteristics with existing medical insights, could help elucidate the disease’s pathology and inform therapeutic decision-making.

There are several limitations to this study. First, our study did not include essential information regarding iodine exposure levels, TPO antibody status with respect to thyroid disease, and histopathological findings. Iodine intake may be a potential confounder in our results; moreover, anti-TPO antibodies are significant in confirming the onset of chronic thyroiditis. Additionally, we did not explore the correlation between imaging and histopathological findings. Accordingly, there is a need for further research that includes these aspects to enhance the comprehensiveness and accuracy of our findings. Second, this single-center study only included one ethnic group. In addition, our screening program is a fee-based examination that includes a wide variety of test items; as a result, the surveyed population may be biased toward economically affluent and health-conscious individuals. Several studies have reported differences in TSH level, CT density, and thyroid volume according to ethnicity, region, diet, and other factors [13,30,35,36,37,38]. Therefore, it is unclear whether or not the present findings are generalizable to other populations. Third, the subsequent course in the case group could not be evaluated. In this study, only screening program data were available; therefore, some important information, such as thyroid antibody levels and subsequent needs for treatment, could not be ascertained. Future studies are required to perform time-series analysis, including information on antibodies, which may provide additional information. Forth, we did not consider lesions within the thyroid gland. Our system evaluated the entire thyroid gland, and thus internal lesions were not excluded from the calculation of the volume and CT densities. The influence of small lesions, including cysts and nodules, on the course of the disease should be investigated separately.

## 5. Conclusions

A high CT density and smaller thyroid volume at baseline are associated with future TSH elevation. The case group experienced a substantial decrease in thyroid CT density, while the control group had a minor decrease, with both groups maintaining a stable thyroid volume over time.

## Figures and Tables

**Table 1:** Characteristics of study population in baseline analysis.

	Case Group (*n* = 86)	Control Group (*n* = 1071)	*p*-Value
Follow-up time (Years) (Median [IQR])	2.1 [1.9–3.4]	5.5 [5.4–5.7]	N/A
Sex (No.)			0.22
Male	51	712	
Female	35	359	
Age (Years) (Median [IQR])	61 [54–69]	56 [48–64]	**<0.01**
TSH (IU/mL) (Median [IQR])	2.80 [2.23–3.63]	1.30 [0.87–1.84]	**<0.01**
T4 (ng/dL) (Median [IQR])	1.08 [0.92–1.23]	1.12 [1.12–1.26]	**<0.01**
CT density (HU) (Median [IQR])	89.8 [74.3–99.8]	81.7 [73.5–90.0]	**<0.01**
Volume (mL) (Median [IQR])	13.0 [10.1–16.4]	15.3 [11.9–19.2]	**<0.01**

IQR, interquartile range. *p*-values < 0.05 are shown in bold.

**Table 2:** Odds ratios of new-onset TSH elevation.

	Non-Adjusted Odds Ratio (95%CI)	*p*-Value	Multivariable-Adjusted Odds Ratio * (95% CI)	*p*-Value
CT density (+10 HU)	1.40 (1.16–1.67)	**<0.01**	1.44 (1.17–1.77)	**<0.01**
Volume (−3 mL)	1.26 (1.10–1.45)	**<0.01**	1.33 (1.11–1.59)	**<0.01**

CI, confidence interval; TSH, thyroid-stimulating hormone. * Estimated from logistic regression models. Multivariable model was adjusted for sex, age, TSH, and T4 at baseline. *p*-values < 0.05 are shown in bold.

**Table 3:** Changes in CT densities and volumes in the case and control groups.

	Baseline	End of Follow-Up *	*p*-Value	Changes
TSH				
Case group	2.80 [2.23–3.63]	5.21 [4.89–5.76]	**	2.53 [1.80–3.25]
Control group	1.30 [0.87–1.84]	1.32 [0.92–1.85]	**	0.02 [−0.27–0.35]
T4				
Case group	1.12 [1.00–1.26]	1.09 [0.99–1.21]	**<0.01**	−0.03 [−0.17–0.11]
Control group	1.08 [0.92–1.23]	1.05 [0.87–1.17]	**0.04**	−0.04 [−0.23–0.11]
CT density				
Case group	89.8 [74.3–99.8]	77.5 [66.0–90.7]	**<0.01**	−8.4 [−17.1–−1.1]
Control group	81.7 [73.5–90.0]	78.3 [71.0–86.5]	**<0.01**	−2.9 [−8.5–1.8]
Volume				
Case group	13.0 [10.1–16.4]	13.1 [9.9–16.3]	0.23	−0.1 [−1.9–1.2]
Control group	15.3 [11.9–19.2]	15.1 [12.0–18.9]	0.29	−0.1 [−1.4–1.2]

The values are presented as medians (interquartile ranges). CT, computed tomography; TSH, thyroid-stimulating hormone. * When the TSH level exceeded the upper limit for the case group and at the visit from 5 to 6 years after the initial visit for the control group. ** TSH is a variable with an arbitrary cutoff; hence, *p*-values were not calculated. *p*-values < 0.05 are shown in bold.

**Table 4:** Linear regression results for the impact of changes in CT on variations in TSH levels.

	Coefficient	Standard Error	95%CI	*p*-Value
Constant	0.124	0.034	0.058–0.190	**<0.01**
Changes in CT (HU)	−0.022	0.003	−0.029–−0.015	**<0.01**

*p*-values < 0.05 are shown in bold.

## Data Availability

The data presented in this study are available on request from the corresponding author. The data are not publicly available due to informed consent not presupposing the individual data’s public availability.
